# Dietary Patterns, Dietary Interventions, and Mammographic Breast Density: A Systematic Literature Review

**DOI:** 10.3390/nu14245312

**Published:** 2022-12-14

**Authors:** Elisa Pastore, Saverio Caini, Benedetta Bendinelli, Domenico Palli, Ilaria Ermini, Nora de Bonfioli Cavalcabo’, Melania Assedi, Daniela Ambrogetti, Miriam Fontana, Giovanna Masala

**Affiliations:** 1 Clinical Epidemiology Unit, Institute for Cancer Research, Prevention and Clinical Network (ISPRO), Via Cosimo il Vecchio 2, 50139 Florence, Italy; 2 Cancer Risk Factors and Lifestyle Epidemiology Unit, Institute for Cancer Research, Prevention and Clinical Network (ISPRO), Via Cosimo il Vecchio 2, 50139 Florence, Italy; 3 Breast Cancer Screening Branch, Institute for Cancer Research, Prevention and Clinical Network (ISPRO), Via Cosimo il Vecchio 2, 50139 Florence, Italy

**Keywords:** diet, a priori dietary patterns, a posteriori dietary patterns, dietary intervention, mammographic breast density, observational studies, randomized controlled trials, systematic review

## Abstract

Background: Breast cancer (BC) is the most common and deadliest malignancy among women. High mammographic breast density (MBD) is an established modifiable risk marker for BC, and it is of interest, for prevention purposes, to consider lifestyle factors that may modulate both MBD and BC risk. Here, we conducted a systematic review of the most up-to-date evidence on the association between diet as a whole and MBD. Methods: We considered as eligible for inclusion in our review (PROSPERO registration code CRD42022335289) the studies published until 31 December 2021, that reported on the association between a priori or a posteriori dietary patterns (in observational studies) or dietary interventions (in randomized controlled trials) and MBD. Results: In total, twelve studies were included. MBD tended to be inversely associated with adherence to dietary patterns characterized by high consumption of plant-based foods and low in meat, animal fats, and alcohol, defined both a priori (e.g., Mediterranean diet and WCRF/AICR guidelines) or a posteriori (e.g., “fruit-vegetable-cereal” and “salad-sauce-pasta/grains” patterns). Findings from intervention studies were in fair agreement with those from observational studies. Conclusions: While further studies are needed, we found suggestive evidence that the adoption of a healthy diet is associated with lower MBD.

## 1. Introduction

Breast cancer (BC) is the most common and deadliest malignant tumor among women worldwide. According to data from the Global Cancer Observatory (GCO), over 2.25 million new cases of BC were diagnosed, and 684,996 BC-related deaths occurred in 2020 [1].

Many of the factors that modulate BC risk are related to exposure to endogenous hormones or genetic predisposition and are therefore not easily targeted by primary prevention; however, some potentially modifiable risk factors have also been identified. Among these, mammographic breast density (MBD), defined as the proportion of fibroglandular breast tissue over the whole breast volume, is an established, strong, and independent risk marker for BC development [2]. There is by now convincing evidence that women with dense breasts have a higher risk of developing BC compared to those with low-density breasts [3,4].

Over the years, several studies focused on MBD and its association with personal characteristics that are also associated with BC risk. Lower body mass index (BMI), younger age, nulliparity, and late age at first child are directly associated with MBD among both pre-menopausal and post-menopausal women and hormone replacement therapy among post-menopausal women. Moreover, smoking and education are inversely associated with MBD among pre-menopausal women [5].

Of particular interest for prevention purposes are those factors that are potentially modifiable (e.g., lifestyle habits) and modulate MBD and BC risk in the same direction. Physical activity is a well-known protective factor for BC [6], but the evidence regarding any association with MBD is inconsistent to date, and a systematic review published in 2012 reported no association [7]. More recently, the DAMA trial, a multi-arm 24-month intervention study performed on 226 post-menopausal women with high baseline MBD, showed a reduction in MBD both in the dietary intervention and in the physical activity intervention arm in comparison with controls [8].

Concerning diet, many studies evaluated in recent years the association between MBD and specific beverages, foods or food groups (e.g., alcohol, vegetables, fruits, and meat), or nutrients (e.g., vitamin A, calcium, and others), with mostly inconclusive results except for alcohol intake [9,10]. Indeed, a recent meta-analysis of 11 studies [11] showed that there was a direct association between MBD and alcohol consumption, which is also an established risk factor for BC [12]. Far fewer studies have been carried out that examined the link between diet as a whole (in randomized controlled trials (RCTs) or in observational studies by means of dietary patterns) and MBD. Unlike focusing on single foods and food groups, examining ones’ dietary style in its entirety has the advantage of taking into account that several foods are frequently consumed in combination and that the association of specific foods also extends to the nutrients they contain, which might exert synergistic effects on disease prevention and treatment.

A number of new studies that examined the association between diet as a whole and MBD have appeared in recent years, so we considered it timely and appropriate to conduct a systematic review with the aim of updating previous evidence [13] and gaining a deeper understanding of whether and how MBD could be used as a potentially modifiable biomarker in BC prevention strategies.

## 2. Materials and Methods

### 2.1. Literature Search and Inclusion Criteria

We conducted the literature search according to the PRISMA 2020 guidelines for meta-analyses of observational studies [14], and we registered the protocol in the Prospective Register of Systematic Reviews (PROSPERO), with registration code CRD42022335289 [15]. The literature search was conducted on 31 December 2021, in *PubMedMEDLINE* and *EMBASE*. In order to minimize the risk of missing eligible articles, the bibliographic search was conducted by using a string designed to be as sensitive as possible and specifically: (diet OR dietary OR food OR alcohol OR alcoholic OR energy) AND (“breast density” OR “breast volume” OR “dense area” OR mammography OR mammographic OR mammogram). Of note, the search string did not make explicit reference to either dietary patterns or dietary intervention: the articles using the latter as exposure were selected out of all those that focused on the association between any aspect related to diet and MBD, thus ensuring higher sensitivity. After removing duplicates, we initially discarded papers on the basis of title and abstract in order to retain only those potentially of interest for our review, which were then read in full text to assess their eligibility. We searched for additional eligible papers in the reference list of selected articles and previously published literature reviews.

We considered as eligible the articles that reported on the association of a priori or a posteriori dietary patterns (in observational studies) or dietary intervention (in randomized clinical trials) with MBD or other measures of breast composition (e.g., dense and non-dense area). The studies that reported on the relationship between dietary patterns or dietary intervention and BC risk only (i.e., not considering MBD) were excluded. In case of studies yielding results from the same cohort or study population, we considered them as independent studies as long as they reported novel results (e.g., MBD in relation to different dietary patterns). The literature search and study selection were carried out independently by two researchers (M.F. and E.P.), and any disagreement was resolved by consensus with a senior researcher (S.C.).

### 2.2. Data Extraction

The following information was extracted from all eligible papers: first author and year of publication; study design, country in which the study was conducted, and name of the study; size of the whole study population and its distribution in terms of demographics (i.e., sex and age) and menopausal status; methods used for data collection on food consumption (e.g., food-frequency questionnaires (FFQs), dietary records, or dietary recalls); type of dietary exposure: a priori or a posteriori dietary patterns (for observational studies, either with a cross-sectional or a prospective design) or dietary intervention (for RCTs); and summary of results (measure of association, 95% confidence intervals (CI), and *p*-values or any other available measure of statistical uncertainty if those were not provided) for the whole study sample and stratified for menopausal status and other variables of interest if available (if unadjusted and adjusted results were available, the latter were considered). The data extraction and organization into tables was conducted by two researchers (M.F. and E.P.) and independently checked by a third author (S.C.) to ensure that everything was correct.

We had initially planned to conduct meta-analysis (using random effects models) to pool study-specific results into summary estimates and corresponding 95% CI and to quantify heterogeneity across studies using the I^2^ statistics (and to use meta-regression and subgroup analysis to identify sources of variation in case of large heterogeneity). However, as illustrated in more detail below (see Results), the type of dietary exposure evaluated in relation to MBD, the method of MBD assessment, and the statistical analysis methods that were applied all varied widely across articles. Due to the small number of studies and their heterogeneity, it was eventually not possible to conduct a formal meta-analysis of the available evidence as according to the initial plans. In the Results section, the studies included in the review are therefore summarized and presented according to the strength of scientific evidence: first cross-sectional studies, then cohort studies, and finally, intervention studies.

### 2.3. Quality Assessment and Risk of Bias

The quality assessment and evaluation of the risk of bias for all the studies included in the review were conducted (independently by two investigators, M.L. and E.P.; any disagreement was solved by consensus meeting with a third investigator, S.C.) by using the critical appraisal tools prepared by the Joanna Briggs Institute (JBI) [16]. Different checklists were used for studies having an RCT, cohort, or cross-sectional designs (of note, the latter checklist was used also for those paper in which a cross-sectional analysis was conducted within a study having a prospective design).

## 3. Results

**Figure 1:**
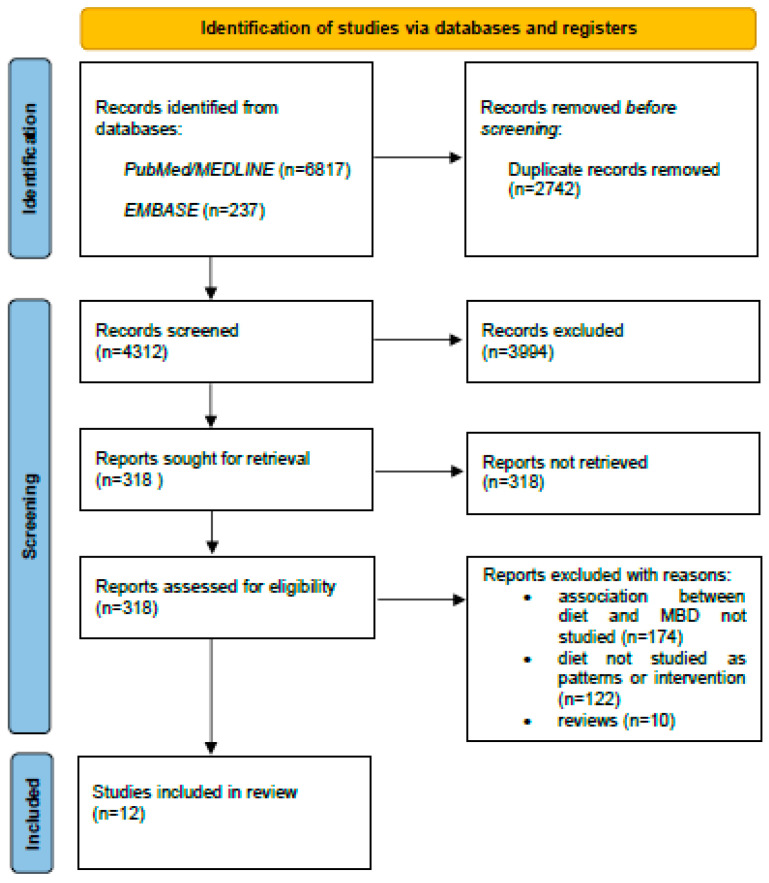
Flow diagram for the literature search and selection of articles on the association between a priori and a posteriori dietary patterns and dietary interventions and mammographic breast density (MBD).

The literature search returned over 7000 entries, of which 4312 were non-duplicate articles (Figure 1): of these, 3994 were discarded based on their title and abstract, leaving 318 to be read in full text. A further 174 articles were discarded for not reporting any findings on the association between diet and MBD, and 10 previously published reviews were also removed. Of the remaining 134 articles, 122 studied how MBD was affected by the intake of single foods, beverages, or nutrients, and only 12, which were eventually included in the present review, were independent reports on the association between a priori or a posteriori dietary patterns (observational studies) or dietary interventions (RCT) and MBD (Figure 1).

### 3.1. Cross-Sectional Studies

Six studies evaluated the relationship between the adherence to dietary patterns, defined as a priori or a posteriori and MBD, using a cross-sectional analysis (in a few cases, nested within studies with different designs, e.g., cohort or nested case-control studies).

The “Determinants of Mammographic Density in Spain” (DDM-Spain) study was a multicenter, cross-sectional investigation involving pre- and post-menopausal women aged 45–69 years and attending breast cancer screening programs [17]. The study participants completed a FFQ during the preceding year before having their screening mammogram. First, dietary data were used by the study investigators in the aim to quantify the compliance with the WCRF/AICR (World Cancer Research Fund/American Institute for Cancer Research) lifestyle recommendations for cancer prevention, and the latter was then studied in its association with MBD (assessed by a radiologist). A higher compliance with the WCRF/AICR guidelines was linked with lower MBD among post-menopausal women (n = 2734; OR 0.91, 95% CI 0.84–0.99, for 1-unit increment) and non-smokers (n = 2180, OR 0.87, 95% CI 0.80–0.96, for 1-unit increment), while among pre-menopausal women and smokers, the inverse association failed to achieve statistical significance (Table 1 and Table S1).

In a later paper, the DDM-Spain research group reported on the relationship between adherence to two a-posteriori-defined dietary patterns (Western and Mediterranean) and radiologist-assessed MBD [18]. These two dietary patterns were derived by applying principal components analysis (PCA) to dietary data collected in a previous case-control study [23]. The Western dietary pattern was based on a high intake of high-fat dairy products, refined grains, and processed foods (processed meat, sweets, caloric drinks, convenience food, and sauces) and a low intake of low-fat dairy products and whole grains. The Mediterranean pattern was characterized by a high consumption of fish and plant-based foods (fruits and vegetables, pulses, boiled potatoes, olives, and vegetables oil) and a low consumption of juices [24]. Multivariable analyses revealed that women with higher adherence to the Western dietary pattern had higher MBD: the OR was 1.25 (95% CI 1.03–1.52) when comparing the women in the 4th vs. 1st quartiles of adherence and 1.09 (95% CI 1.02–1.18) for one standard deviation increase (Table 1). In stratified analysis, this association held only among women with BMI > 25 kg/m^2^ (Supplementary Table S1). No associations emerged between adherence to the Mediterranean dietary pattern and MBD.

The effect of the Mediterranean diet on MBD was investigated in two additional studies. Voevodina et al. [19] conducted a cross-sectional study in Ulm, Germany, that involved pre- and post-menopausal women aged 21–84 years, who completed a FFQ about their current diet at the same time as they underwent mammography. In age- and BMI-adjusted analyses, a higher Mediterranean diet score was inversely associated with radiologist-assessed MBD among post-menopausal women (n = 271, OR 0.94, 95% CI 0.89–0.99 for 1-unit increment) and non-smokers (n = 369, OR 0.95, 95% CI 0.90–0.99 for 1-unit increment) (Table 1 and Table S1). Further adjustment confirmed the direction and magnitude of the association between Mediterranean diet score and MBD. Apart from the well-known limitations of cross-sectional studies (i.e., the lack of a time interval between the assessment of the exposure and of the outcome of interest), which of course affect all of the studies reviewed in this section, the main limitation of the DDM-Spanish study and of the investigation by Voevodina et al. was that MBD was not measured by means of objective criteria (e.g., automated software) but assessed by radiologists (Supplementary File S1).

Tseng et al. [20] cross-sectionally examined the link between a Mediterranean Diet Scale (MDS) and MBD among pre- and post- menopausal women who were recruited into the Minnesota Breast Cancer Family Study. The study participants filled out an FFQ about their diet over the past year and provided a recent mammogram, from which radiologists estimated MBD by a semi-automated method. In fully adjusted models conducted in the whole study sample, no associations were observed between MDS and MBS, but analysis stratified by smoking status revealed that MDS was inversely associated, with statistical significance, with MBD among current smokers (n = 176, β −1.68, *p*-value 0.002, for 1-unit increment; β −7.17, *p*-value 0.01, for highest vs. lowest category) (Table 1 and Table S1). Upon modifying the MDS by adding 1 to women who did not consume alcohol (vs. women who consumed 5–25 g/day), the inverse association between MDS and MBD resulted stronger in the whole study sample although still not achieving statistical significance and also among current smokers (β moved to −1.90, *p*-value 0.0005, for 1-unit increment; and to −8.07, *p*-value 0.004, for highest vs. lowest category).

The same group of investigators also examined the association between MBD and three a posteriori patterns (emerged from principal component analysis (PCA) on data obtained by a 153-item FFQ) within the Minnesota Breast Cancer Family Study [21]. Regression analysis showed that the “fruit-vegetable-cereal” pattern was inversely associated with MBD (close to statistical significance: β −0.13, *p*-value 0.09) among pre-menopausal women (Table 1). Further analysis stratified by smoking status showed significant (or nearly significant) inverse associations between the “fruit-vegetable-cereal” and the “salad-sauce-pasta/grain” patterns with MBD among current smokers only (β −0.30, *p*-value 0.02; and β −0.27, *p*-value 0.06, respectively) (Supplementary Table S1). Except for the inherently bias-prone cross-sectional design, the investigations conducted by Tseng et al. within the Minnesota Breast Cancer Family study did not suffer from any major limitations (Supplementary File S1).

Finally, Takata et al. [22] conducted a cross-sectional analysis within a case control nested within the Hawaii component of the Multiethnic Cohort, including both pre- (n = 303) and post-menopausal (n = 947) women. Three a posteriori dietary patterns were identified by using factor analysis from data obtained by means of a FFQ: “fat and meat”, “vegetables”, and “fruit and milk”. No statistically significant difference was found between any of these three food patterns and MBD (assessed by a radiologist using a computer-assisted method and modelled in quartiles) (Table 1). In subgroup analysis, higher scores in the “fat and meat” pattern were positively associated with higher MBD except among pre-menopausal women. The largest difference in MBD between women in the 4th and 1st quartiles scores of the “fat and meat” pattern were observed among women of Japanese ethnicity (38.1% vs. 34.3% *p* for trend = 0.22). Further analysis stratified by case/control status did not substantially alter the results (Supplementary Table S1).

### 3.2. Cohort Studies

Of all the studies included in this review, only two assessed the association between dietary patterns (either a priori or a posteriori) and MBD using a cohort study design (Table 2). In both studies, both pre- and post-menopausal women were included and separately analyzed.

Garzia et al. [25] examined the effects of two different inflammatory-associated dietary patterns (a pro-inflammatory and an anti-inflammatory one, based on the Alternative Healthy Eating Index—AHEI) on pre-menopausal MBD, measured from screening mammograms using the Cumulus software (Table 2). The study population included control participants from a case-control study nested within the Nurses’ Health Study II (NHSII). Participating women first completed a FFQ about their current diet at 27–44 years old (Adult-FFQ), and then, when they were 33–52 years old, they filled out another FFQ about their diet during high school (HS-FFQ). No significant association was found between the adherence to either dietary pattern in adolescence or early adulthood and MBD in multivariable-adjusted models.

A birth cohort study by Mishra et al. [26] evaluated the association between MBD, measured at a mean age of 51.5 years using the Cumulus software, and diet, measured at two different points in life: at four years of age by means of 24 h recalls, from which three a posteriori patterns were derived using PCA, and in adulthood, measured by means of 5-day food records, from which four a posteriori patterns were derived (also using PCA). Regression analysis detected no association between childhood dietary patterns and MBD among 792 women who were pre- or post-menopausal at mammographic examination. Regarding the diet recorded in adulthood, higher adherence to the “alcohol and fish” and “high fat and sugar” patterns was associated with higher MBD (β 0.08, CI 95% 0.01–0.15; and β 0.07, CI 95% 0.00–0.14) in models unadjusted by energy intake. However, the strength of the association was attenuated, and statistical significance was lost in energy intake-adjusted regression models (Table 2).

The cohort study by Garzia et al. did not have any major limitation, while in the study by Mishra et al., there was a lack of clarity about any strategies that were put in place to deal with confounding factors during data analysis (Supplementary File S1).

### 3.3. Intervention Studies

Four articles were identified that evaluated the effects of dietary interventions at some point in life on MBD by using an RCT design (Table 3).

The DAMA (Diet, physical Activity and MAmmography) study was a 24-month intervention trial that involved 234 healthy post-menopausal women (aged 50–69 years at enrolment) with MBD > 50% as measured within the local mammographic screening program in Florence [27]. The DAMA study had a 2 × 2 factorial design and implemented two interventions (dietary and physical activity); thus, the participants were randomly allocated to one of four study arms (dietary intervention, physical activity intervention, both interventions, or control group). The dietary intervention consisted of a gradual change towards a diet mainly based on plant food, with low glycemic load, low in saturated- and trans-fats and alcohol, and rich in antioxidants. Women assigned to the control group were given general advice on healthy dietary patterns according to the WCRF recommendations issued in 2007. Information on dietary habits was collected both at study baseline and study end by means of a validated FFQ that was previously used in the European Prospective Investigation into Cancer and Nutrition (EPIC) [28]. By fully exploiting the factorial design, the primary objective of the DAMA study was to assess the association of either intervention with changes in MBD between the study baseline and the study end (measured on digital mammograms using a fully automated software). A difference-in-difference statistical analysis was conducted. The dietary intervention was found to be effective in reducing MBD: at the end of the study, a reduction of 9% in MBD (95% CI 3–14) was observed among women who underwent the dietary intervention compared to the control group after taking into account baseline MBD [8] (Table 3). The main limitation of the DAMA study, which was shared by the other RCTs included in this review (and is in fact inescapable given the nature of the intervention) lies in the fact that the participants could not be made blind to the arm of assignment, and therefore, some contamination (represented by women who were not randomized to the intervention but who nonetheless engaged themselves in changing their dietary habits according to the WCRF recommendations) cannot be ruled out (Supplementary File S1).

Dorgan and colleagues conducted a prospective investigation of 182 pre-menopausal women (aged 25–29 years old) who had previously participated in the Dietary Intervention Study in Children (DISC) at 8–10 years of age [29]. The DISC was a multicenter RCT designed to evaluate the efficacy of a specific dietary intervention in children in reducing the elevated serum low-density lipoprotein cholesterol (primary endpoint). The “usual care group” received educational materials about heart-healthy eating patterns. The intervention was initially planned to last 3 years and later extended until the participants reached a mean age of 16.7 years. In the follow-up study, the investigators evaluated the long-term effects of the cholesterol-lowering dietary intervention on MBD as measured with customized image processing software. The diet of the study participants at the time of MBD assessment was also assessed by means of three 24 h dietary recalls. No significant differences in MBD were observed by treatment group in unadjusted or adjusted analyses (Table 3). In addition to the aforementioned impossibility of concealing the outcome of the randomization to the participants, in the study by Dorgan et al., it was unclear whether the participants were analyzed in the group to which they had been randomized (Supplementary File S1).

Boyd et al. conducted a randomized controlled trial involving 817 women (mean age 64.8 years) with high MBD at baseline with the aim of determining whether a two-year dietary intervention, characterized by a low-fat, high-carbohydrate isocaloric diet, could reduce MBD (the control group received no specific dietary advice) [30]. MBD was measured by study radiologists at baseline and two years after randomization. Dietary assessment was carried out by multiple interviews conducted by trained dietitians: once a month in the intervention group and once every four months in the control group during the first year and every three months in both groups during the second year. At the time of the interviews, subjects also had to provide a three-day dietary records. Women in the intervention group had a non-significant reduction in MBD by −0.21% (95% CI −0.95 to 0.52, *p*-value 0.57) compared to controls after adjusting for age, menopausal status, and weight change between the baseline and the end of the study (Table 3). Because post hoc analyses suggested that the effect of the dietary intervention on MBD might be mediated by menopausal status, Martin and colleagues replicated the same analyses in another group of high-MBD women (participating in the same ongoing trial but separate from those included in the study by Boyd et al.) with a longer follow-up time (mean duration 4.0 years instead of 2.3 years) [31]. Participants were all pre-menopausal at the beginning of the study and became post-menopausal during follow-up. The intervention lasted two years and consisted of an isocaloric diet, with the specific aims of reducing fat intake to 15% of total energy intake and increasing carbohydrate intake to cover at least 65% of total energy intake. The dietary assessment was carried out by dietitians once a month in the first year, quarterly in the second year, and twice a year in the following years among the intervention group and quarterly, twice a year, and annually among the control group. MBD was measured at baseline and after menopause by means of a computer-assisted method. Women were divided into those who had become post-menopausal within two years of randomization (n = 189) and those who had become post-menopausal at least two years after randomization (n = 272). The average distance in time between the first and the second mammogram was 2.4 and 5.0 years in the former and the latter group of participants, respectively. The change in MBD was −3.8% in the intervention group vs. −6.2% in the control group (*p*-value 0.06, unadjusted analysis) among women who became post-menopausal within two years of randomization and −11.3% in the intervention group vs. −11.1% in the control group (*p*-value 0.84, unadjusted analysis) among women who became post-menopausal ≥2 years after randomization (Table 3), thus confirming that menopausal status could act as an effect modifier for the impact of a dietary intervention on MBD. The main limitations were the lack of an explicit statement about whether the outcome assessors were blind to the treatment assignment for the study by Boyd et al. and about whether the follow-up was complete and the analysis was conducted according to an intention-to-treat approach in the study by Martin et al. (Supplementary File S1).

**Table 3:** Main characteristics and results (for the whole study group or stratified by menopausal status when available) of randomized controlled studies that investigated the effect of dietary intervention on mammographic breast density.

Author, Year	Study Country (and Name)	Study Population	Dietary Assessment	Dietary Intervention	Summary of Results ^(a)^
Masala et al., 2019 [27]	DAMA, Italy	Post-menopausal, high MBD (n = 234, mean age 58.6 y, SD 5.6)	FFQ filled at baseline and study end (24 months after the enrolment)	Diet based on plant food, low glycemic load, low in saturated- and trans-fats and alcohol, rich in antioxidants; duration 2 years.	MBD ratio 0.91 (0.86–0.97) for intervention vs. control, *p*-value 0.003
Dorgan et al., 2010 [29]	DISC (Dietary Intervention Study in Children), USA	Prepubertal girls (intervention arm: n = 118, mean age 9.2 y, SD 0.6 at randomization and 27.3 y, SD 1.0 at MBD assessment; control arm: n = 112, mean age 9.2 y, SD 0.6 at randomization and 27.2 y, SD 1.1, at MBD assessment)	Three 24 h dietary recalls.	Diet low in total and saturated fat and cholesterol intake and rich in dietary fiber (fruits, vegetables, whole grains).	MBD 19.7% (17.0–22.7) for intervention vs. 18.3% (15.9–21.0) for control, *p*-value 0.51
Martin et al., 2008 [31]	Canada	Pre-menopausal, high MBD (intervention arm: n = 93, mean age 48.7 y, SD 3.2; control arm: n = 96, mean age 48.6 y, SD 2.8), who became post-menopausal prior to 2 y post randomization.	Dietary records and dietary interviews: - First year: monthly in intervention group, every 4 month in control group - Second year: every 3 months in intervention group, every 6 months in control group - Subsequent years: twice a year in intervention group, once a year in control group	Isocaloric low-fat, high-carbohydrate diet (15% of calories from fat, 20% from protein, 65% from carbohydrates); duration 2 years.	Change in MBD: −3.8% (intervention) vs. −6.2% (control), *p*-value 0.06
		Pre-menopausal, high MBD (intervention arm: n = 124, mean age 48.7 y, SD 3.2; control arm: n = 272, mean age 48.6 y, SD 2.8), who became post-menopausal at least 2 y post randomization.			Change in MBD: −11.3% (intervention) vs. −11.1% (control), *p*-value 0.84
Boyd et al., 1997 [30]		Pre-menopausal, high MBD (n = 817, mean age 46.8 y, SD NA)	Dietary records and dietary interviews: - First year: monthly in intervention group, every 4 months in control group - Second year: every 3 months in both groups		Change in MBD: −0.21% (−0.95 to 0.52) for intervention vs. control, *p*-value 0.57

^(a)^ Most adjusted results and corresponding 95% confidence intervals were reported whenever available. FFQ, food-frequency questionnaire; MBD, mammographic breast density; NA, not available; SD, standard deviation.

## 4. Discussion

We conducted a systematic literature review of observational and intervention studies focusing on the association between dietary style and MBD, with the ultimate goal of gaining a deeper understanding of the potential of diet-based interventions for BC prevention. Specifically, we were interested in the whole diet consumed by individuals as opposed to specific foods and food groups: accordingly, we restricted our review to studies that considered a priori and a posteriori dietary patterns or dietary interventions in their association with MBD. A total of twelve studies were included in our review.

Albeit with due caution dictated by the limited number of available studies and the imperfect consistency of their results, it appears that dietary patterns inversely associated with MBD tend to share some similarities, including a high consumption of cereals, vegetables, fruits, and vegetable oil and a low intake of saturated and trans fats, red meat, processed foods, and alcohol. Conversely, diets that include a high consumption of red meat, high-fat dairy products, sweet foods and beverages, and alcohol tended to be associated with higher MBD. These data are consistent with the evidence on the association between single food components and MBD. Particularly, a greater consumption of vegetables and/or olive oil (rich in mono-unsaturated fats) was found to be independently associated with lower MBD in a large Italian longitudinal study [10] and two cross-sectional studies conducted in the USA and Spain [32,33]. With regard to animal saturated fats, a recent cross-sectional study [34] evaluated the association between low- and high-fat dairy foods intake and MBD in 1546 women who underwent breast cancer screening mammography at two private clinics in Canada: the results showed that a greater consumption of high-fat and low-fat dairy foods was, respectively, associated with higher and lower MBD, particularly in pre-menopausal women. Whole-milk intake was also directly associated with MBD in one of the studies previously mentioned [33], while a negative association between MBD and cheese or dairy products emerged in two other abovementioned studies [10,32]. Since adolescence is the period of life when the greatest breast tissue development occurs, Bertrand et al. [35] studied the influence of adolescent animal fat intake on pre-menopausal MBD in the Nurses’ Health Study II (NHSII) women; the researchers found a significant positive association between animal fat intake (mainly from red meat, milk and dairy products, and chicken) and MBD. These results are consistent with the findings reported by Tseng et al. [36], who highlighted a significant direct association between red meat consumption in adolescence and adult MBD measured in a group of Chinese immigrant women. Among the studies included in this review, only a few evaluated to what extent MBD in adulthood is influenced by exposure to specific dietary patterns during adolescence and mostly detected no associations; thus, longitudinal, suitably sized, and methodologically sound studies seem to be warranted in order to answer this research question. Finally, regarding sweet foods and beverages, a cross-sectional study [37], which analyzed data from the same study population examined by Canitrot et al. [34], showed that an increase in consumption of sweet food and sugar-sweetened beverages was associated with higher MBD in the whole study group and, respectively, post-menopausal women. These results are consistent with the observation that dietary patterns rich in fat and sugar were found to be directly associated with MBD in some of the studies that were included in our review [17,26]. By and large, the above findings strongly suggest that some specific dietary components may be more effective (compared to others included in the same dietary pattern/style) in affecting MBD. However, it should be noted that some conflicting results also exist: for example, Voon et al. performed a cross-sectional study among Malaysian women and found a positive association between consumption of mutton, pork, vegetables, sweets, snacks, soy bean, and eggs and MBD, while no relationship was detected for grains, meat, beverages, oil, and fruits [9].

A possible explanation of the inconsistencies emerging across studies focusing on single foods in relation with MBD could lie in the fact that while individual foods may exert a beneficial role on disease prevention, they are generally consumed within a meal along with other foods (e.g., vegetables may be frequently consumed with oil but also red meat, thus making it difficult to disentangle the effect of each food on MBD because of collinearity). Furthermore, each food contains nutrients that can influence the absorption of other nutrients, with either synergistic or inhibitory effects [38]. The assessment of dietary patterns (e.g., the Mediterranean diet, the adherence to WCRF recommendations, and others) may circumvent these limitations and represent a more adequate scientific approach, as it allows to capture overall information on dietary habits, including consumption of foods, which could act as protective or risk factors in cancer prevention as well as their interactions.

The findings from our review and of most other studies (briefly summarized in the previous paragraphs) focusing on single foods are also consistent with studies that examined the link between diet and BC risk. In fact, several studies underlined how the Mediterranean dietary pattern also exerts protective effects with respect to BC risk. Specifically, Schwingshackl et al. performed a systematic review and meta-analysis of available literature in 2017 [39] in order to study the effects of adherence to a Mediterranean diet on cancer risk and cancer mortality [10]. Based on the results from seventeen studies (all of which were observational studies except one RCT), the researchers concluded that the highest adherence score to a Mediterranean diet was inversely associated with BC risk. Later, these results were confirmed in a multi-centric case-control study conducted in six Italian areas and in the Canton of Vaud in Switzerland [40].

A controversial component of the Mediterranean diet (as well as other dietary patterns) is alcohol, which is considered as a class I carcinogen in humans by IARC [41], and it is recognized as a strong and modifiable risk factor for BC even when consumed at low levels [42]. Indeed, the issues about alcohol may relate to the total lifetime amount, the period of intake in relation to breast tissue development (e.g., between menarche and menopause and during pregnancy, when breast tissue is most susceptible to proliferation), and the general patterns of alcohol consumption, and several possible mechanisms have been put forward through which alcohol may increase BC risk [43,44]. Several studies investigated the association between alcohol intake and MBD, mostly reporting positive associations, although with mixed results. For example, the cross-sectional study by Voevodina et al. reported a direct association between consumption of more than 10 g/day of alcohol and MBD in pre- and post-menopausal women, while two other studies reported no association in pre- and post-menopausal women for intakes of about 2–3 drinks/week [45] or in post-menopausal women for intakes >90 g of alcohol per week [46]. Based on the above, studies evaluating diet and MBD should consider alcohol as a potential confounder and take appropriate countermeasures when designing the study or during data analysis. Among the articles included in our review, some studies did not evaluate alcohol intake at all [29,30,31], while others make an adjustment during data analysis [17,19,20,25]. In the study by Mishra et al., the “alcohol and fish” pattern was positively associated with MBD in models not adjusted by energy intake, while the study by Takata et al. did not include alcohol in the patterns, but the results did not change by adjusting the analysis for alcohol intake. It deserves further consideration the fact that the Mediterranean diet (and thus the Mediterranean scores) includes a small amount of daily alcohol intake (between 5 g and 25 g per day), often in the form of red wine. In this regard, Masala et al. found a negative association between MBD and low-alcohol diet in the dietary intervention arm of the study; the adherence of WCRF recommendations examined by Castellò et al. included limited alcohol consumption (less than 1 drink/day); and in the study by Tseng et al., the inverse association between the MDS and MBD was strengthened after removing the alcohol component. This might suggest that a varied diet rich in plant-based foods and antioxidants might be able to counteract, at least to some extent, the harmful effect of alcohol on breast tissue.

Most findings on changes in MBD in relation to lifestyle components come from cross-sectional studies, while prospective studies (either observational or RCT) are scarce and not always consistent in their results, so it is difficult to determine how long it takes to modify MBD by means of changes in one’s lifestyle and what are the most susceptible periods in life for these changes to occur. Furthermore, most existing RCTs focus on single elements of one’s diet and lifestyle (e.g., soy isoflavones supplementation [47,48,49], aerobic exercise [50], or green tea extracts [51]). The aforementioned DAMA study is the only RCT, to our knowledge, that simultaneously considered the effect of changes in dietary patterns and physical activity levels on MBD, and its findings that a significant reduction in MBD may be achieved by a 2-year intervention among post-menopausal women suggests that even short interventions are worth being put in practice, as they have a not-negligible impact on an important risk factor for BC development (in addition to their well-known beneficial impact on countless other aspects of health).

The main strength of the present paper lies in our having reviewed and updated the available evidence on the association between dietary patterns or interventions and MBD, which was particularly well-timed given that large-sized epidemiological studies (both observational and experimental in design) have been recently published on this topic [17,18,23,27]. (Moreover, we also included an earlier study [26] that was not considered in previous reviews [13,52].) Dietary patterns provide a snapshot of the diet as a whole and are more stable over time than the consumption of individual components of the diet. Moreover, as already mentioned, assessing diet as a whole has the advantage of taking into account combinations of specific foods, which in turn contain nutrients with possible synergistic effects [53]. Of note, the methodological quality of the studies was judged as being generally very good, which reassures on the reliability of the results that could be drawn from this review. Our work has some weaknesses that are important to consider, which (as usual in this kind of endeavor) are partly the consequences of limitations affecting the individual studies that are included in the review. In particular, we were unable to conduct a formal meta-analysis, as we had initially planned, due to the small number of available studies and their substantial heterogeneity, which extended to the following key aspects: (a) differences between dietary patterns; (b) timing of collection of information on dietary patterns (e.g., in adulthood, during childhood, or during adolescence); (c) differences in the distance in time between assessment of dietary patterns and MBD measurement; (d) methodological differences in the mode of MBD detection and classification; (e) differences in statistical analysis methods and criteria of stratification; and (f) different methods of dietary assessment (FFQ, 24 h recalls, food diaries). Moreover, the number of studies eventually included in the review was rather limited despite the large number of those that were returned by the literature search in *PubMed* and *EMBASE* (over 7000, which is due to the high sensitivity of the search string). While acknowledging that the amount of research on a given topic is limited is a legitimate and valuable output of a literature search, having more studies eligible for inclusion in the present review would have allowed us to draw much firmer conclusions.

## 5. Conclusions

In conclusion, we found suggestive evidence that a healthy diet (i.e., a diet based primarily on vegetables, fruit, cereals, and legumes and low in animal-based foods, saturated fats, alcohol) is inversely associated with MBD, which is, in turn, an established risk marker for BC development. While the overall evidence is fairly consistent with that from studies focusing on single foods and foods groups and with studies focusing on the link between dietary patterns and BC risk, it should be acknowledged that our conclusions are based on a rather limited number of studies, most of which have a cross-sectional design. Thus, evidence from prospective investigations (both observational, i.e., cohort studies, and RCT) is still required in order to corroborate these preliminary findings. Moreover, prospective studies in which the link between diet, MBD, and BC risk is investigated longitudinally, possibly by also taking advantage of repeated measurements taken at different points in one’s lifetime, would further help disentangle the role of MBD in mediating the effect of diet and other personal characteristics and lifestyle aspects on BC risk. While conducting more research in this field is therefore warranted in order to validate the currently sparse findings and bridge existing knowledge gaps (e.g., whether an interaction exists with menopausal status and other behavioral risk factors for BC development such as cigarette smoking), this review confirms that the adoption of a healthy diet can play an essential role in containing the BC burden at population level and should be promoted on every occasion.

## Figures and Tables

**Table 1:** Main characteristics and results (for the whole study group or stratified by menopausal status when available) of cross-sectional studies reporting on the association between a-priori- or a-posteriori-defined dietary patterns and mammographic breast density.

Author, Year	Study Country (and Name)	Study Population	Dietary Assessment	Dietary Pattern Type	Dietary Pattern	Summary of Results ^(a)^
Castellò et al., 2016 [17]	DDM-Spain	Pre- and post-menopausal (n = 3548, mean age 56.2 y, SD = 5.5 y)	FFQ (filled at 45–69 y)	A priori	Western dietary pattern	OR 1.25 (1.03–1.52) for 4th vs. 1st quartiles
						OR 1.09 (1.02–1.18) for 1 -SD increment
					Mediterranean dietary pattern	OR 0.99 (0.81–1.21) for 4th vs. 1st quartiles
						OR 1.02 (0.95–1.09) for 1 SD increment
Castellò et al., 2015 [18]	DDM-Spain	Pre-menopausal (n = 816, mean age 49.8 y, SD = 2.9 y)	FFQ (filled at 45–69 y)	A priori	WCRF/AICR recommendations	OR 0.91 (0.56–1.50) for highest vs. lowest scores
						OR 0.97 (0.84–1.13) for 1-unit increment
		Post-menopausal (n = 2734, mean age 58.1 y, SD = 4.5 y)				OR 0.77 (0.59–1.01) for highest vs. lowest scores
						OR 0.91 (0.84–0.99) for 1-unit increment
Voevodina et al., 2013 [19]	Germany	Pre-menopausal (n = 150, <50 y)	FFQ (filled at 21–84 y)	A priori	Mediterranean Diet Score	OR 0.99 (0.89–1.10) for 1-unit increment
		Post-menopausal (n = 274, ≥50 y)				OR 0.94 (0.89–0.99) for 1-unit increment
Tseng et al., 2008 [20]	Minnesota Breast Cancer Family Study, USA	Pre- and post-menopausal (n = 1286, mean age 57 y, SD = 12 y)	FFQ	A priori	Mediterranean Diet Scale	β −0.54 for highest vs. lowest category, *p*-value 0.56
						β −0.27 for 1-unit increment, *p*-value 0.17
					Revised Mediterranean Diet Score	β −1.40 for highest vs. lowest category, *p*-value 0.15
						β −0.33 for 1-unit increment, *p*-value 0.09
Tseng et al., 2008 [21]	Minnesota Breast Cancer Family Study, USA	Pre-menopausal (n = 356, mean age 57 y, SD = 11.8 y)	FFQ	A posteriori	Fruit-vegetable-cereal	Mean MBD 28.3% vs. 31.2% for 5th vs. 1st quintile
						β −0.13 for 1-unit increment, *p*-value 0.09
		Post-menopausal (n = 930, mean age 57 y, SD = 11.8 y)				Mean MBD 19.2 vs. 19.4 for 5th vs. 1st quintile
						β 0.03 for 1-unit increment, *p*-value 0.37
		Pre-menopausal (n = 356, mean age 57 y, SD = 11.8 y)			Salad-sauce-pasta/grain	Mean MBD 28.2 vs. 30.3 for 5th vs. 1st quintile
						β −0.10 for 1-unit increment, *p*-value 0.26
		Post-menopausal (n = 930, mean age 57 y, SD = 11.8 y)				Mean MBD 19.9 vs.18,2 for 5th vs. 1st quintile
						β 0.04 for 1-unit increment, *p*-value 0.48
		Pre-menopausal (n = 356, mean age 57 y, SD = 11.8 y)			Meat-starch	Mean MBD 33.2 vs. 30.0 for 5th vs. 1st quintile
						β 0.07 for 1-unit increment, *p*-value 0.40
		Post-menopausal (n = 930, mean age 57 y, SD = 11.8 y)				Mean MBD 20.4 vs. 19.5 for 5th vs. 1st quintile
						β 0.03 for 1-unit increment, *p*-value 0.55
Takata et al., 2007 [22]	Multiethnic Cohort Study, Hawaii, USA	Pre-menopausal (n = 303, mean age 59.7 y, SD = 8.8 y)	FFQ at cohort entry	A posteriori	Vegetables	Mean MBD 42.1% vs. 39.7% 4th vs. 1st quartile, *p*-trend 0.27
		Post-menopausal (n = 947, mean age 59.7 y, SD = 8.8 y)				Mean MBD 30.0% vs. 30.6% 4th vs. 1st quartile, *p*-trend 0.71
		Pre-menopausal (n = 303, mean age 59.7 y, SD = 8.8 y)			Fruit and milk	Mean MBD 42.5% vs. 42.1% 4th vs. 1st quartile, *p*-trend 0.94
		Post-menopausal (n = 947, mean age 59.7 y, SD = 8.8 y)				Mean MBD 30.7% vs. 30.4% 4th vs. 1st quartile, *p*-trend 0.80
		Pre-menopausal (n = 303, mean age 59.7 y, SD = 8.8 y)			Fat and meat	Mean MBD 41.9% vs. 42.3% 4th vs. 1st quartile, *p*-trend 0.89
		Post-menopausal (n = 947, mean age 59.7 y, SD = 8.8 y)				Mean MBD 31.7% vs. 29.3% 4th vs. 1st quartile, *p*-trend 0.23

^(a)^ Most adjusted results odds ratio (OR, for logistic regression models), or â coefficients (for linear regression models), along with corresponding 95% confidence intervals (CI) and/or *p*-values, were reported whenever available. Lacking this information, we reported the mean/median MBD in the groups being compared, along with the *p*-values for comparison (if available). DDM-Spain, Determinants of Mammographic Density in Spain; FFQ, food-frequency questionnaire; MBD, mammographic breast density; SD, standard deviation; WCRF/AICR, World Cancer Research Fund/American Institute for Cancer Research. y = years.

**Table 2:** Main characteristics and results (for the whole study group, or stratified by menopausal status when available) of cohort studies reporting on the association between a-priori- or a-posteriori-defined dietary patterns and mammographic breast density.

Author, Year	Study Name and Country	Study Population	Dietary Assessment	Dietary Pattern Type	Dietary Pattern	Summary of Results ^(a)^
Garzia et al., 2021 [25]	NHS-II, USA	Pre-menopausal (n = 1117, median age 44 y)	Adult-FFQ (diet in early adulthood, filled at 27–44 y)	A priori	Pro-inflammatory pattern	Mean MBD 40.7% (38.6–42.8) vs. 40.6% (38.6–42.7) for the 5th vs. 1st quintile, *p*-value for trend 0.99
					AHEI anti-inflammatory pattern	Mean MBD 39.9% (37.9–42.0%) vs. 39.7% (37.7–41.7%) for the 5th vs. 1st quintile, *p*-value for trend 0.98
		Pre-menopausal (n = 709, median age 44 y)	HS-FFQ (diet during high school, filled at 33–52 y)		Pro-inflammatory pattern	Mean MBD 38.7% (35.7–41.7%) vs. 39.6% (37.3–41.9%) for the 5th vs. 1st quintile, *p*-value for trend 0.93
					AHEI anti-inflammatory pattern	Mean MBD 41.4% (38.8–43.9%) vs. 41.0% (38.3–43.6%) for the 5th vs. 1st quintile, *p*-value for trend 0.89
Mishra et al., 2011 [26]	The Medical Research Council National Survey of Health and Development, UK	Pre- and post-menopausal (n = 792, mean age 51.5, SD = 1.1 y)	Maternal 24 h recalls (about diet at 4 y)	A posteriori	breads and fats	β −0.004 (−0.08, 0.07)
					fried potatoes and fish	β −0.05 (−0.12, 0.01)
					milk, fruit and biscuits	β −0.01 (−0.08, 0.05)
		Pre- and post-menopausal (n = 700, mean age 51.5, SD = 1.1 y)	5-day food diaries (about diet at 36 and 43 y)		low fat, high fiber	β 0.03 (−0.04, 0.11)
					alcohol and fish	β −0.02 (−0.13, 0.17)
					high fat and sugar	β 0.06 (−0.01, 0.13)
					meat, potatoes, and vegetables	β −0.03 (−0.10, 0.04)

^(a)^ Most adjusted results odds ratio (OR, for logistic regression models), or â coefficients (for linear regression models), along with corresponding 95% confidence intervals (CI) and/or *p*-values, were reported whenever available. Lacking this information, we reported the mean/median MBD in the groups being compared, along with the *p*-values for comparison (if available). AHEI, Alternative Healthy Eating Index; FFQ, food-frequency questionnaire; MBD, mammographic breast density; NHS-II, Nurses’ Health Study II; SD, standard deviation.

## Data Availability

Not applicable.

## Supplementary Materials

The following supporting information can be downloaded at: https://www.mdpi.com/article/10.3390/nu14245312/s1, Table S1: Results of analyses stratified by body mass index (BMI), smoking status, and ethnicity, in the studies reporting on the association between a priori- or a posteriori-defined dietary patterns and mammographic breast density; File S1: Quality assessment and risk of bias of the studies included in the review.
