# Supplementary material for: Dietary Patterns, Dietary Interventions, and Mammographic Breast Density: A Systematic Literature Review
Source: Nutrients. 2022 Dec 14;14(24):5312. doi: 10.3390/nu14245312 (PMC9781545; doi:10.3390/nu14245312)
Supplement: Supplementary file 1 [file nutrients-14-05312-s001.zip › Supplementary Table S1.pdf]

Supplementary Table S1. Results of analyses stratified by body mass index (BMI), smoking status, and ethnicity, in the studies reporting on the association between *a priori*- or *a posteriori*-defined dietary patterns and mammographic breast density.

Author, year	Study population	Dietary pattern	Summary of results [a]
Castellò, 2016[17]	BMI < 25 (n=1015)	Adherence to Western dietary pattern (a priori)	OR 0.98 (0.70 - 1.39) for 4th vs.1st quartiles
	BMI ≥ 25 (n=2533)		OR 1.41 (1.13 - 1.76) for 4th vs.1st quartiles
Castellò, 2015[18]	Non smoker or former +6 months (n=2180)	Compliance with the WCRF/AICR recommendations (a priori)	OR 0.64 (0.47 - 0.87) for highest vs. lowest scores<br>OR 0.87 (0.80 - 0.96) for 1-unit increment
	Smoker or former <6 months (n=1370)		OR 1.05 (0.73 - 1.49) for highest vs. lowest scores<br>OR 1.01 (0.91 - 1.12) for 1-unit increment
Voevodina, 2013[19]	Smoker (n=51)	Mediterranean dietary score (a priori)	OR 0.97 (0.80 - 1.19) for 1-unit increment
	Non-smokers (n=369)		OR 0.95 (0.90 - 0.999) for 1-unit increment
Tseng, 2008[20]	Non-smokers (n=1110)	Mediterranean Diet Scale (a priori)	β 0.13 for highest vs. lowest category, p-value 0.90<br>β -0.08 for 1-unit increment, p-value 0.72
	Current smokers (n=176)		β -7.17 for highest vs. lowest category, p-value 0.01<br>β -1.68 for 1-unit increment, p-value 0.002
	Non-smokers (n=1110)	Revised Mediterranean Diet Score (a priori)	β -0.58 highest vs. lowest category, p-value 0.59<br>β -0.12 for 1-unit increment, p-value 0.58
	Current smokers (n=176)		β -8.07 for highest vs. lowest category, p-value 0.004<br>β -1.90 for 1-unit increment, p-value 0.0005
Tseng, 2008[21]	Current smoker (n=176)	Fruit-vegetable-cereal (a posteriori)	mean MBD 25.0% vs. 31.4% for 5th vs. 1st quintile<br>β -0.30 for 1-unit increment, p-value 0.02

	Non-smokers (n=1110)		mean MBD 23.7 vs. 23.4 for 5th vs. 1st quintile<br>β 0.03 for 1-unit increment, p-value 0.48
	Current smoker (n=176)	Salad-sauce-pasta/grain (a posteriori)	mean MBD 22.4 vs. 26.4 for 5th vs. 1st quintile<br>β -0.27 for 1-unit increment, p-value 0.06
	Non-smokers (n=1110)		mean MBD 24.4 vs.23.4 for 5th vs. 1st quintile<br>β 0.03 for 1-unit increment, p-value 0.48
	Current smoker (n=176)	Meat-starch (a posteriori)	mean MBD 28.4 vs. 24.0 for 5th vs. 1st quintile<br>β 0.14 for 1-unit increment, p-value 0.18
	Non-smokers (n=1110)		mean MBD 25.8 vs. 23.9 for 5th vs. 1st quintile<br>β 0.04 for 1-unit increment, p-value 0.40
Takata, 2007[22]	Caucasians (n=1046)	Vegetables (a posteriori)	mean MBD 34.2% vs. 29.7% 4th vs. 1st quartile, p-trend 0.18
	Japanese (n=1638)		mean MBD 33.7% vs. 38.1% 4th vs. 1st quartile, p-trend 0.13
	Caucasians (n=1046)	Fruit and milk (a posteriori)	mean MBD 33.1% vs. 32.5% 4th vs. 1st quartile, p-trend 0.74
	Japanese (n=1638)		mean MBD 37.0% vs. 37.8% 4th vs. 1st quartile , p-trend 0.61
	Caucasians (n=1046)	Fat and meat (a posteriori)	mean MBD 31.9% vs. 29.9% 4th vs. 1st quartile, p-trend 0.84
	Japanese (n=1638)		mean MBD 38.1% vs. 34.3% 4th vs. 1st quartile, p-trend 0.22

(a) Most adjusted results and corresponding 95% confidence intervals were reported whenever available. WCRF/AICR: World Cancer Research Fund/American Institute for Cancer Research. MBD: mammographic breast density.
